# Severe Symptomatic Anemia in Gastrointestinal Tract Sarcoidosis

**DOI:** 10.7759/cureus.44867

**Published:** 2023-09-07

**Authors:** Jin Tao, Gabriela S. Generette, Myra Khan, Naser Khan

**Affiliations:** 1 Internal Medicine, Mercyhealth Internal Medicine Residency Program, Rockford, USA; 2 Pathology and Laboratory Medicine, Mercyhealth Internal Medicine Residency Program, Rockford, USA; 3 Gastroenterology, Mercyhealth Internal Medicine Residency Program, Rockford, USA

**Keywords:** anemia, chest pain, gastrointestinal disease, sarcoidosis, multiorgan sarcoidosis, gastrointestinal pathology

## Abstract

Sarcoidosis is a systemic granulomatous disease of unknown etiology with the potential to involve many organs of the body. Less than 1% of patients with sarcoidosis have GI manifestations. Here, we report a case of GI tract sarcoidosis that presented with severe symptomatic anemia.

A 51-year-old female with a history of pulmonary and liver sarcoidosis presented to the emergency room with a one-week history of chest pain and shortness of breath. A physical exam was significant for conjunctival pallor. On admission, her hemoglobin was 6.9 g/dL. Her iron studies showed anemia of chronic disease. There was no evidence of recurrent pulmonary sarcoidosis on the CT scan of the chest. Transthoracic echo showed no abnormal wall motion movements. A nuclear stress test was negative for perfusion defects. She underwent esophagogastroduodenoscopy (EGD) and colonoscopy to further evaluate potential sources of active GI tract blood loss. Biopsies of gastric mucosa and small bowel revealed non-caseating granulomas. Immunohistochemical stains for acid-fast bacilli and fungus were negative. Random biopsies of erythematous mucosa from the colonoscopy were unremarkable. The patient’s history of pulmonary and liver sarcoidosis along with non-caseating granulomas found in the gastric mucosa and small bowel suggest GI tract sarcoidosis manifestations. She was started on corticosteroids with complete resolution of symptoms in five months.

Clinical presentation varies widely based on the specific organ involvement, as well as the underlying pathophysiology of the organ damage. The pathogenesis of sarcoidosis is poorly understood and attributable to both genetic and environmental factors. Overall, the treatment of sarcoidosis is not standardized. It is primarily driven by the effect of sarcoidosis on the patient’s symptoms and quality of life. However, symptomatic sarcoidosis usually responds well to corticosteroids. We believe that clinicians should maintain a high level of vigilance for patients with a known history of sarcoidosis and new symptoms, as these might signal sarcoid involvement of a new organ and help guide the diagnostic and treatment process.

## Introduction

Sarcoidosis is a systemic granulomatous disease of unknown etiology with the potential to involve many organs of the body. GI tract involvement is estimated to occur in less than 1% of patients with sarcoidosis [1]. GI tract sarcoidosis presents with a wide range of symptoms and varies according to the organs involved [2]. Here, we report a rare case of GI tract sarcoidosis that presented with severe symptomatic anemia.

## Case presentation

A 51-year-old female with a past medical history of hypertension, type 2 diabetes, end-stage kidney disease on peritoneal dialysis, and iron deficiency presented to the emergency room with a one-week history of chest pain and shortness of breath. The chest pain was described as stabbing pain and numbness sensation under her left breast with associated shortness of breath. Two days prior, she started feeling dizzy with mild abdominal cramping. She denied dysuria, hematuria, hematemesis, hematochezia, or melena. Her medications included amlodipine 10 mg QD, metoprolol 25 mg BID, insulin glargine 10 units HS, and insulin lispro 5 units with meals.

The patient had similar left-sided chest pain eight years ago. CT scan then revealed a 16 x 13 mm left upper lobe nodule. PET scan showed an increase in metabolic activity as well as a hypermetabolic liver lesion concerning for metastasis. She then underwent a left thoracotomy that demonstrated the lung nodule to be a mediastinal lymph node. The biopsy was diagnostic for non-caseating granulomas and suspicious for sarcoidosis. Fungal and acid-fast bacilli cultures performed were negative. Serology for antinuclear antibody, anti-neutrophilic cytoplasmic antibody, and Histoplasma titers were within normal limits. At that time, she was treated with prednisone which improved her symptoms with residual chest pain.

**Figure 1:**
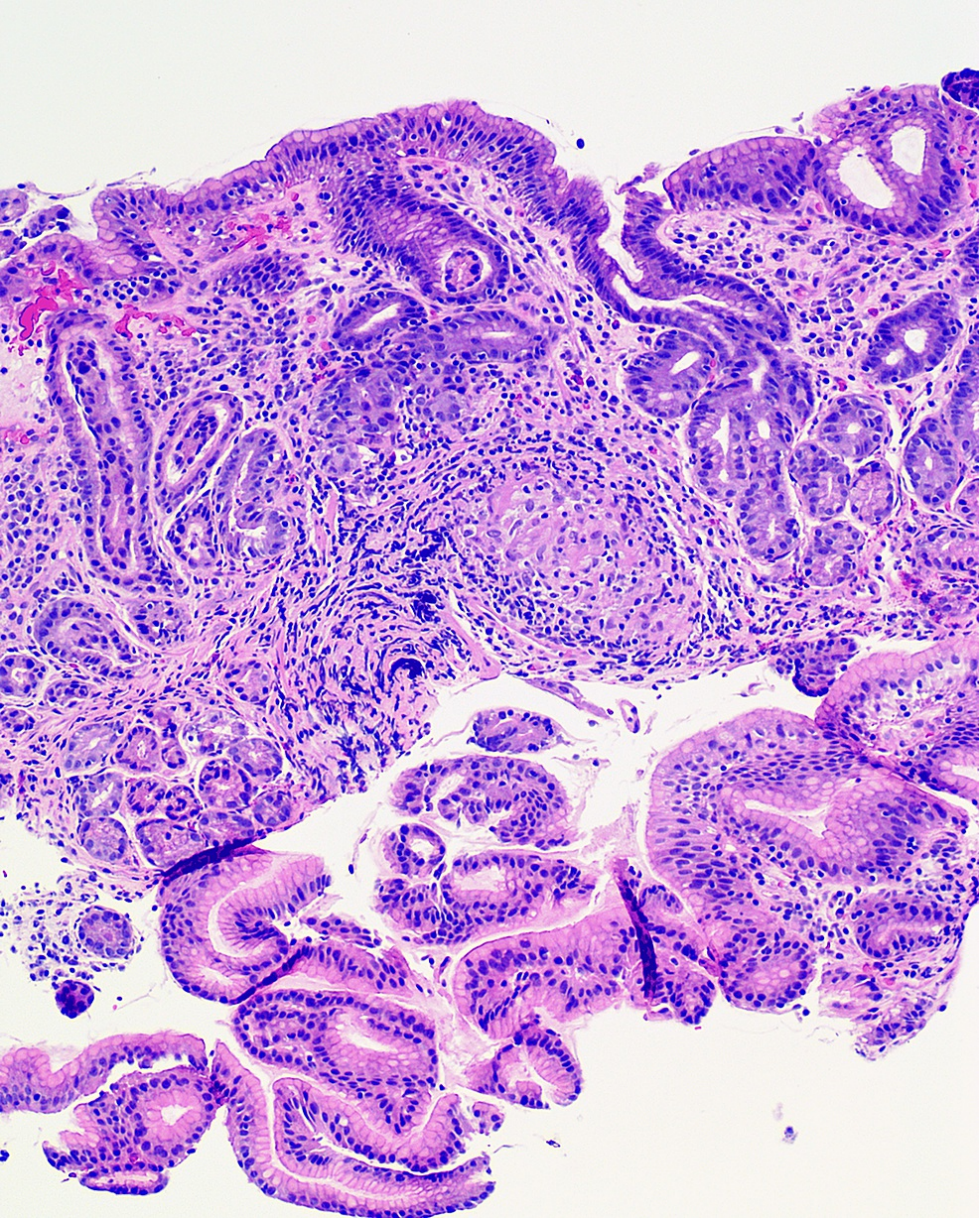
Stomach (high magnification): one noncaseating granuloma made up of a mix of lymphocytes, plasma cells, and epithelioid histiocytes involving the stomach lamina propria

Upon admission, her physical exam was remarkable for conjunctival pallor and positive digital rectal exam. Labs showed hemoglobin of 6.9 gm/dL. Iron studies showed iron 83 mcg/dL (37-145 mcg/mL), total iron binding capacity 188 mcg/dL (251-346 mcg/mL), and ferritin 554.0 ng/mL (13.0-150.0 ng/mL) suggesting anemia of chronic disease. Troponins trended 143-167-163 ng/mL and were elevated at baseline in the setting of her ESRD. Creatinine was 4.9 mg/dL which was her baseline. EKG was normal with no ST or T wave changes compared to prior. There was no evidence of recurrent pulmonary sarcoidosis on the CT scan of the chest. Transthoracic echo showed no abnormal wall motion movements and grade 1 diastolic dysfunction. The nuclear stress test was negative for perfusion defects. She underwent esophagogastroduodenoscopy (EGD) and colonoscopy to further evaluate potential sources of active GI tract blood loss. Biopsies of gastric mucosa and small bowel revealed non-caseating granulomas (Figure 1). Immunohistochemical stains for acid-fast bacilli and fungus were negative for microorganisms. Random biopsies of erythematous mucosa from the colonoscopy were unremarkable. The patient’s history of pulmonary and liver sarcoidosis along with non-caseating granulomas found in the gastric mucosa and small bowel suggest GI tract sarcoidosis manifestations. She was started on corticosteroids with hemoglobin improved to 10 gm/dL and complete resolution of symptoms in five months.

## Discussion

Involvement of the GI tract in sarcoidosis is extremely rare. The largest published literature on this topic analyzed 305 cases of GI involvement [2]. GI involvement of sarcoidosis may be the first presentation of the systemic disease or it may occur in patients with known sarcoidosis. The most commonly involved portion of the GI tract is the stomach, particularly the antrum. Sarcoidosis has also been known to appear in the esophagus, small intestine, appendix, colon, and rectum [3].

Clinical presentation varies widely based on the specific organ involvement, as well as the underlying pathophysiology of the organ damage [2]. The pathogenesis of sarcoidosis is poorly understood and attributable to both genetic and environmental factors. A key role in the development of sarcoidosis is played by T cells as they promote cellular immune reaction and are usually associated with an inverted CD4/CD8 ratio. It is well characterized by noncaseating granuloma typically containing macrophages, multinucleated giant cells, and epithelioid cells. Both the tumor necrosis factor (TNF) and TNF receptor are elevated in this disease [4].

Dysphagia is the main presenting symptom of the esophageal sarcoidosis. Its nature has been attributed to multiple mechanisms, such as mucosal or muscular layer infiltration and involvement of the myenteric plexus or extrinsic compression [5-6]. With gastric sarcoidosis, the clinical presentation is usually related to the presence of peptic ulceration or narrowing of the gastric lumen by granulomatous inflammation. It commonly presents as epigastric pain, nausea, and vomiting [7]. The small bowel is less frequently involved in GI sarcoidosis. Symptoms range from diarrhea and abdominal pain to small bowel obstruction and hemorrhage [8]. The colon and rectum are rarely the site of sarcoidosis and usually do not have any clinical manifestations [9].

Regardless of the organ involvement, sarcoidosis of the GI tract may present as anemia [9] such as in our patient. She had symptomatic anemia manifesting in chest pain. Her digital rectal exam was positive which led to further investigations of the GI tract as a bleeding source. The pathology findings from gastric mucosa and small bowel revealed non-caseating granulomas in the lamina propria with multinucleated giant cells which are typical findings of sarcoidosis granuloma [10]. Histology findings, however, are only a part of the evidence on which the diagnosis of sarcoidosis is based [11]. The other two components are the exclusion of other causes of granulomatous disease and clinical, radiographic, and histopathologic evidence of sarcoidosis in at least one other organ [12]. In our patient, the differential is broad, and it includes Crohn’s disease, Whipple disease, tuberculosis, fungal infections, syphilis, foreign body reaction, and *Histoplasma capsulatum* enteritis [13,14]. Investigation for potential infectious and non-infectious causes was unremarkable in our case. However, it is particularly problematic to distinguish sarcoidosis from Crohn’s disease, as the features of these two diseases overlap [15]. GI sarcoidosis is most often asymptomatic, though, while Crohn’s disease presents most commonly with diarrhea [15]. Granulomas are seen in both, but crypt inflammation, ulcers, and aphthae are more suggestive of Crohn’s disease [16]. In addition, the detection of granulomas outside the GI tract would favor sarcoidosis, just as it did in our patient.

Asymptomatic and non-progressive disease requires no treatment, as a majority of patients undergo spontaneous remission. However, in general, symptomatic extrapulmonary sarcoidosis such as GI tract sarcoidosis is usually treated with 20-40 mg of daily prednisone as the initial dose. Then, the corticosteroid should be tapered to the lowest effective dose. Patients usually respond well to corticosteroids and are treated for at least one year [17]. Additional anti-sarcoid therapies such as methotrexate, azathioprine, and cyclophosphamide could also be considered depending on the specific type of organ involvement. Overall, the treatment of sarcoidosis is not standardized. It is primarily driven by the effect of sarcoidosis on the patient’s symptoms and quality of life [17].

## Conclusions

Despite 305 cases of GI sarcoidosis reported, this clinical entity still remains a rarity. Anemia related to GI sarcoidosis has previously been described; however, there was no case reported, where it would have such a severe presentation as in our patient. We believe that clinicians should maintain a high level of vigilance for patients with a known history of sarcoidosis and new symptoms, as these might signal sarcoid involvement of a new organ and help guide the diagnostic and treatment process.
